# Food Industry Views on Pulse Flour—Perceived Intrinsic and Extrinsic Challenges for Product Utilization

**DOI:** 10.3390/foods11142146

**Published:** 2022-07-20

**Authors:** Rie Sadohara, Donna M. Winham, Karen A. Cichy

**Affiliations:** 1 Department of Plant, Soil and Microbial Sciences, Michigan State University, East Lansing, MI 48824, USA; sadohara@msu.edu (R.S.); karen.cichy@ars.usda.gov (K.A.C.); 2 Department of Food Science and Human Nutrition, Iowa State University, Ames, IA 50011, USA; 3 Sugarbeet and Bean Research, USDA-ARS, East Lansing, MI 48824, USA

**Keywords:** flour performance, research and development, food industry, flour quality, plant-based food, bean flour

## Abstract

Pulses such as beans, chickpeas, peas, and lentils are typically consumed whole, but pulse flours will increase their versatility and drive consumption. Beans are the most produced pulse crop in the United States, although their flour use is limited. To expand commercial applications, knowledge of pulse flour attributes important to the food industry is needed. This research aimed to understand the food industry’s needs and barriers for pulse flour utilization. An online survey invitation was sent via direct email to individuals employed in food companies developing wheat flour products. A survey weblink was distributed by pulse commodity boards to their membership. Survey questions asked food manufacturers about intrinsic factors of pulse flours that were satisfactory or challenging, and extrinsic factors for use such as market demand. Of the 75 complete responses, 21 currently or had previously used pulse flours in products, and 54 were non-users of pulse flours. Ten users indicated that there were challenges with pulse flours while five did not. Two of the most selected challenges of end-product qualities were flavor and texture. Over half of the respondents were unfamiliar with bean flour. Increasing awareness of bean flours and their attributes coupled with market demand for pulse flour-based products may be the most important extrinsic factors to increasing use among food manufacturers rather than supply or cost.

## 1. Introduction

Pulses are dry seeds of starchy leguminous crops cultivated worldwide, serving as a source of protein, dietary fiber, vitamins, and minerals [1,2]. The most produced pulses include common bean, pea, chickpea, cowpea, and lentil, and their global production is over 71 million tons (5-year average of 2016–2020) [3]. In addition to their nutritive values, evidence has been accumulating that pulses provide protective effects against cardiometabolic diseases and certain types of cancer [4,5]. Due to their health-promoting effects, a weekly consumption of 1.5 cups of pulses is recommended by the Dietary Guidelines for Americans for a 2000-kcal diet [6]. In addition, pulses are a key component of sustainable diets. Like other legumes, pulses demonstrate biological nitrogen fixation which reduces dependency on commercial fertilizers [7,8,9]. About 2.9 million tons of common bean, pea, chickpea, and lentil are produced in the U.S. annually (5-year average of 2016–2020) [3]. However, whole pulse consumption is low in the U.S., with about 20% of adults reporting consumption of dry, canned, or frozen pulses in the previous 24 h [10]. Barriers to pulse utilization include limited time for meal preparation, gastrointestinal discomfort, and lack of knowledge on dry pulse cooking [11,12,13,14]. Pulse flours (milled pulses) offer solutions to some of the barriers, as they can be processed into a broader range of ready to eat or quick to prepare food products, such as baked goods, snacks, pasta, and extruded products [15,16]. The high levels of protein and dietary fiber (15–30% each) in pulses make their flours an ideal ingredient to increase nutritional quality of traditionally wheat-flour based products [4,15]. Wheat flour alternatives are gaining attention, especially pea and chickpea flours, due to increasing consumer interest in the nutritional and health benefits of pulse flours [17,18]. In fact, pulse flour, starch, or protein were listed 1666 times as ingredient(s) in food products launched in the U.S. in 2016–2020, and peas accounted for 65% of these [19].

Numerous studies have been conducted to investigate the suitability of pulse flours for various food applications with various substitution ratios to wheat flour [20,21,22]. Pulse flour utilization brings some challenges such as off- and ‘beany’ flavors and product texture. Functional properties of pulse flours are different from wheat flour due to the lack of gluten [22,23,24]. Thakur et al. [25] compared pulse and wheat flours with established milling quality specifications. They assert that pulse flour description and seed quality parameters for pulse milling need to be defined for industry consistency. A lack of studies on the effect of genotype, environment, storage, processing, and milling on those quality parameters, as well as flour components and their interactions, was noted [25]. Performance can vary depending on pulse flour and application types, and flours can be characterized by many attributes, such as dehulling efficiency, physicochemical properties, thermal behavior, and nutrient composition [21,26,27,28]. End-product qualities, such as appearance, aroma, and flavor, are also important to consider for consumer acceptability.

The perspectives of end-users on pulse flours (i.e., the food industry) are scarce in the literature. One case study, conducted among European and multinational food manufacturers of pulse-based products, identified important factors for pulse-based product development [29]. Those factors were grouped into three categories: technical aspects, market trends, and ingredient availability. Technical aspects included the functionality and behavior of pulse flours when processed. As expected, the difference between regular wheat flour and pulse flours was noted. While dependent upon time and country, this research found that market trends were strong drivers for company product development. Wary consumer attitudes towards new foods were deemed to be a challenge for pulse-based pasta in Europe, although they were more accepted in the U.S. Ingredient availability was a concern, especially to firms with large production capacities. Since this study was conducted almost exclusively with firms in Europe, a similar study with U.S. food manufacturers is needed to facilitate an improved understanding of the pulse flour-related challenges more specific to the U.S.-based firms. In addition, important pulse flour traits depend on application types. Therefore, optimum pulse flour traits need to be specified for each type of application.

Common bean (*Phaseolus vulgaris*, L.) is the most produced pulse crop globally and in the U.S. [3] Approximately one million tons of dry beans are harvested annually and consumed primarily in the form of canned beans in the U.S. [30] However, beans can also be milled into flour and used for food products that are typically made from wheat flour. Bean flour has been tested for various food applications on a laboratory scale, such as quick bread [31,32], tortillas [33] pasta [34,35,36], cookies [24], biscuits [37], cakes, crackers, and meat analogues [38], with promising results. Bean flour has been commercially produced and used, but its market presence remains rather small. Among the aforementioned 1666 counts of pulse use in food products, bean flour accounted for only 3% of them [19]. Aroma, appearance, texture, and beany flavor, likely caused by lipid oxidation, may be a challenge [38], not only for bean flour but also for other types of pulse flours [25,39]. This suggests that there is a need to improve beans for use as flour, but exactly which traits do not meet the expectations of users are not clear. Moreover, some pulses, including beans, contain high levels of lectins, which can be toxic to the human digestive system when consumed raw but be safe after proper cooking [13]. Knowledge and attitudes about lectins among food manufacturers are an important consideration for improving beans for flour, but relevant information is limited.

Despite some of the possible challenges, their positive impact on sustainability and human health makes pulse flours a promising ingredient for food products. The number of products that utilize pulse flours is rising [18,40]. From breeders’ perspective, however, specific needs for pulse flours by the food industry is an important missing piece of information for developing pulse varieties for flour purposes. This survey study aimed to identify the needs for pulse flours and the barriers limiting the adoption of them by U.S. manufacturers making typically wheat flour-based food products, e.g., pastas, breads, and baked goods.

## 2. Materials and Methods

### 2.1. Ethics Approval

This survey study was deemed exempt under 45 Code of Federal Regulations 46. 104(d) 2ii by the Institutional Review Board at Michigan State University (Study ID: STUDY00002778). Respondents gave consent to participate in the study by clicking “Yes, I agree to participate in this survey”. No compensation for participation was offered or provided.

### 2.2. Survey Development

Survey questions were developed through interviews with academic researchers and bean industry personnel involved in pulse flour research and promotional activities related to dry beans. The interview questions included sections about corporate research and development pipelines and interviewee experience with and their impression of pulse flours. Sixteen flour or milling industry research and development employees were invited to participate in an individual online interview or provide written responses to questions. Fourteen formative interviews were completed by the first author (R.S.). Two participants answered the interview questions by email. A draft survey instrument was developed based on the milling stakeholder responses, information presented at two pulse industry conferences, (Pulse Science and Technology Forum, November 2019; Pulse Flour Summit, March 2020), and informal interviews with conference attendees. The preliminary survey was reviewed for content analysis by 12 academic researchers and food industry personnel who did not participate in the formative interviews. Refinement in wording and question flow was made from their feedback.

### 2.3. Survey Questions for All Participants

#### 2.3.1. User Status, Pulse Flour Type, and Product Types

The survey asked participants about their industry’s current and previous pulse flour use in food products. Pulse flour use response options included: (1) currently using, (2) previously used but not now, (3) have considered using pulse flours, or (4) have never used and do not intend to use pulse flour. “Considered users” refers to industry respondents who have considered using pulses by reviewing research literature or are currently testing pulse flours. The current, previous, and considered (CPC) users were asked about the pulse flour type, and products of interest for utilization or development. Non-users were asked to respond to the same questions hypothetically. For all respondents, if they selected multiple pulse flour types, they were prompted to choose the one most important to them for follow-up questions. “Do not know which pulse flour type is best for my production purpose” was also provided as an option to non-users. The pulse flour types included bean, chickpea, pea, lentil, fava, and other with an option to write in a response. The product types included yeast breads, cakes, quick breads, pastries, cookies, spaghetti, Asian noodles, snack foods, thickening agents, coatings and breading, functional protein products, plant-based meat alternatives, and other with an option to write in a response. The pulse flour type and product type questions required answers to advance in the survey.

#### 2.3.2. Job Titles and Business Types

Questions about the participants and their companies were asked: their primary job titles and business type (single location, headquarters, or branch).

#### 2.3.3. Information, Collaboration, and Impression on Bean Flour

Respondents were asked if they previously searched for technical information on the pulse flour they selected, and if they had, follow-up questions were asked about the sources of information found and the adequacy of this information. They were asked whether they would be interested in providing their opinions to university researchers and plant breeders about how pulse flours can be improved. Respondents were asked to give their specific impressions about bean flour from a list of characteristics. Multiple answers were allowed.

### 2.4. Questions for the CPC Users

#### 2.4.1. Satisfactory and Challenging Characteristics of Pulse Flour-Based Products

The CPC users were asked about product characteristics made from pulse flours that they found satisfactory or challenging, with multiple responses allowed. The choices of product characteristics provided were flavor, mouthfeel, color, texture, appearance, surface smoothness, uniformity, shelf life, mixing properties, dough handling properties, extrusion properties, product yield, volume, size, shape, cooking qualities, mitigating cross-contamination, and other with a comment box. Those terms were to be considered product-specific, and participants were asked to consider those characteristics of the product of their choice. A follow-up question asked the reason(s) for currently using or quitting the use of the pulse flour they selected, with multiple responses allowed.

#### 2.4.2. Variability, Specifications, and Supply

Participants’ opinions were asked with choices “Yes”, “Sometimes”, “No”, and “Do not know” regarding variability in pulse flour quality by batch or supplier, their importance in their product quality, universal specification of pulse flours, gluten contamination, and lectins. Questions were asked with choices “Yes”, “No”, and “Do not know” about cost and supply of the pulse flour they selected. The change in availability of the pulse flour due to the COVID-19 pandemic since March 2020 was asked. All questions included an optional comment box for text entry.

### 2.5. Target Population and Survey Distribution

Qualtrics survey software (Qualtrics ver. March 2021, Provo, UT, USA) was used to develop and distribute the survey regarding pulse flour use. Employee email addresses of food manufacturing firms with one or more of the Standard Industrial Classification (SIC) codes for products typically made from wheat flour (2045, 2051, 2052, 2053, 2096, and 2098) were purchased (Table 1) from InfoUSA Marketing, Inc. (St. Louis, MO, USA). An initial inspection of the email addresses identified seven as duplicates, which were removed.

A direct email invitation was sent to the 12,087 unique addresses purchased. Of these, 1794 emails were identified by Qualtrics as undeliverable. A second invitation email was sent to recipients with a job title considered to be relevant to this study (Table S1) and those who had not started the survey or had partially completed it. The second invitation was sent to 6864 email addresses of which 6741 were valid, and 59 of them at least started the survey. A reminder email was sent 2–7 days after the first and second invitations. In total, 106 responses were collected from the purchased email list. An additional 18 individuals were referred by initial survey invitees or other persons. They were invited directly, and three of them completed the survey.

A second method of data collection by an anonymous URL (weblink) was conducted. Pulse commodity groups (American Pulse Association, US Dry Bean Council, Michigan Bean Commission, USA Dry Pea and Lentil Council, Northarvest), the Institute of Food Technologists chapters in Iowa, and the Great Lakes Section in Michigan distributed the survey link to their members. Faculty members at Iowa State University and Michigan State University Departments of Food Science and Human Nutrition were also included, and the authors distributed the survey weblink to their associates and posted it to their personal Twitter and LinkedIn accounts. A total of 21 responses were collected through the weblink.

## 3. Results and Discussion

Through direct emails and the weblink, 130 responses were collected in total, which included 1 duplicate and 16 declinations. Twenty-nine responses were incomplete, 5 were from outside the U.S., and 4 by businesses irrelevant to wheat or pulse flours. Of the 75 valid responses, 6 were collected via the weblink and 2 via additional invitation emails. Respondents were categorized into four pulse flour user groups for survey question comparisons: 8 current users, 4 previous users, 9 considered users, and 54 non-users. The most common role of the respondents was management or owner (n = 29), followed by research and development (R&D, n = 18). Only 40% (30/75) of the respondents were in R&D or production/chef, who are most likely to be knowledgeable about the behavior of pulse flours when utilized in food products. However, 50% of the current users and 67% of considered users were in R&D, whereas 25% and 13% of the previous and the non-users were in R&D, respectively.

### 3.1. Pulse Flour and Product Types

Participants were asked to choose a specific pulse flour type and product type they would like to produce with the pulse flour they selected (Table 2). Chickpea and pea flours were the most frequently selected as compared to bean, fava, and lentil flours. Sixty-nine percent of non-users did not know which pulse type was best for their needs. Thirteen percent of non-users indicated that they were not interested in using any pulse flours. Over half of ‘considered users’ selected chickpea, whereas 63% of current users and 50% of previous users selected pea flour.

Among all the respondents, yeast breads were the most selected products to use pulse flour for, followed by cookies and quick breads (Table 3). Twenty percent of non-users indicated that they were uninterested in using pulse flours for any products. Though chickpea and pea were the most selected pulse type (Table 2), the type of products using the pulse flours selected varied widely (Table 4). However, yeast breads and cookies (selected nine times each) were relatively popular among those who did not know which type of pulse flour is best for their needs.

### 3.2. Pulse Flour Information

Regarding pulse flour specification or technical information, 75% of the current, 100% of the previous, 78% of the considered users, and 11% of the non-users had looked for technical information on pulse flour. Among those who had looked for information, 61% (14 out of 23) were able to find sufficient information. More CPC users had looked for information on pulse flours than non-users, and 89% of non-users never looked for technical information on pulse flours.

Participants who had looked for information about pulse flours were asked to select all sources of information they consulted. Multiple choices were allowed, and 22 out of the 23 selected at least one source of information. For-profit researchers and industry associations, such as the Institute of Food Technologists, were the most selected source of information (n = 15). Non-profit organizations, such as the American Pulse Association and academic textbooks and researchers, were the second most selected (n = 14). Open-access journals (n = 8) were more popular than subscribed and pay-per-view journals (n = 4). Those who selected “Other” commented that information was obtained from their suppliers (n = 3) and from the Internet (n = 1). From these results, academic and for-profit organizations and communication between researchers seemed to be effective channels likely to reach end-users who sought out information about pulse flours.

### 3.3. Impression on Bean Flour

To gain insights on how beans could be improved for flour purposes, participants were asked for their impressions of bean flour. Multiple answers were allowed. “Do not know about bean flour” was the most selected impression on bean flour, especially by non-users (57%). However, 25–33% of the CPC users also did not know about bean flour (Table 5). Over 60% of the current and the considered users selected “Flavor is a challenge”. All user types selected “Market demand for bean flour is low”. Flavor and functionality were selected as a challenge 22 and 21 times, respectively, whereas gluten contamination and lectin concerns were only selected 6 times in total. Given the low familiarity with bean flour, it is possible that participants were unfamiliar with lectins in beans. A comment mentioned the expected low production volume of bean flour: “Volume limitations limit its commercial usage”. To our knowledge, information about the proportion of beans used commercially as flour in the U.S. is not readily accessible.

### 3.4. Pulse Flour and Product Types of CPC Users

The CPC users were asked the types of pulse flour and the product they are using/used. Yeast breads were the most selected (n = 6), followed by snack foods (n = 5) and plant-based meat alternatives (n = 4) (Table 6). For the eight current users, the most selected reason for using pulse flours was driven by marketing and trends, followed by protein content and functional characteristics of the pulse flour (Table 7). The reason for discontinuing pulse flour by the four previous users were all “Low market demand for the product made with the pulse flour”. No other choices regarding flour performance, supply, or allergen contamination were selected as the reason for discontinuation. These results suggest that market demand is an important determinant on whether or not to use pulse flour. In relation to that, low market demand ranked fourth for bean flour impression (Table 5).

### 3.5. Satisfactory and Challenging Characteristics of Product Quality

In total, 18 out of the 21 CPC users answered the question “Were there challenges when using the pulse flour?” Ten users indicated that they had encountered challenge(s) when using their pulse flour, whereas five indicated they had not (Table 8). Among the 10 respondents who reported pulse flour-related challenges, they produced yeast breads (n = 5), snack foods (n = 2), meat alternatives (n = 2), and cookies (n = 1). In contrast, five respondents indicated there were no challenges for snack foods (n = 2), quick breads (n = 2), and thickening agents (n = 1) with chickpea, pea, and bean flours. It was noteworthy that four respondents used bean and chickpea flours for snack foods, and two reported that there were challenges, whereas the other two reported there were not. It was intriguing that some users in each user status category indicated that there were no challenges and that their product was satisfactory. This implies that it is possible to produce satisfactory quality products for some combinations of pulse flour and product type.

Yeast breads were the main product type that pulse flours were used for, and also pinpointed as the most challenging. This high count may be partly because yeast breads were the most selected products that participants have used or considered using pulse flours for (Table 3). However, the challenges that yeast bread producers reported are useful because gluten formation plays a vital role in yeast bread quality. Gluten is formed during kneading of wheat flour and water, and gluten enables the retention of gas produced during yeast fermentation. The viscoelastic structure of gluten-containing dough leads to high bread volume [41]. In contrast, pulse flours are gluten-free, and thus, they often present challenges in terms of loaf volume and texture when made into breads [41,42]. This survey was designed to characterize the combination of pulse flour and end product as well as the production conditions so that conclusions could be drawn for specific pulse flour types and application. Nevertheless, this was not possible due to the small sample size. Thus, an alternative approach for pulse breeders might be to collaborate with an industry partner to provide feedback on pulse varieties for flours.

The 21 CPC users were asked to select all satisfactory traits of their pulse flour. Texture, appearance, and uniformity were the top three most selected satisfactory characteristics (Table 9). In total, 63% of the current users were satisfied with product color with pulse flours, while only 25% of the previous and 11% of the considered users were satisfied. This suggests the importance of product color for commercial use of pulse flours. Flavor was selected as satisfactory by all user types, which was surprising considering the deemed challenges with off-flavors of pulse flours described as beany, grassy, and bitter [39,43]. This suggests that some food manufacturers know ways to adjust and optimize flavor. Beany flavor may or may not be problematic depending on seasoning used and on the incorporation rate of pulse flours if composite flour is used [38,44]. Unpleasant flavors have also been tackled by a plant breeding approach. A commercially available pea protein ingredient was developed by selecting pea varieties that are low in off-flavors [45].

The 10 respondents who reported there were challenges were asked to select all the product characteristics that they had encountered as issues (Table 10). Flavor, texture, dough handling properties, mouthfeel, and volume were the most selected challenges. Though flavor was the fourth most selected satisfactory characteristic (Table 9), 8 out of the 10 respondents selected flavor as a challenge. While texture was the most selected satisfactory characteristic, 6 out of the 10 respondents selected it as a challenge. Texture is a blanket word that encompasses various attributes (e.g., firmness, brittleness, resilience) depending on product types [46,47,48]. Thus, individual research would be needed to address challenges regarding the texture of specific products. As discussed above, dough handling properties and volume are recognized challenges due to the lack of gluten in pulse flours and the resultant lack of or diluted gluten in the dough [23,29,41]. Three of the four votes to “Dough handling properties” as a challenge were associated with yeast breads. Similarly, three of the four votes to “Volume” were associated with yeast breads. Mixing properties were selected by three CPC users who produced snacks, yeast breads, or meat alternatives. Mixing properties include mixing time and consistency measured during dough-forming [42,49], and desirable mixing properties may differ for those different end products. A blank comment box was provided to each participant to describe the challenges, but only one left a brief comment about “undesirable taste and texture”. Similar to texture, an individual case study would identify challenges specific to product type. The results obtained from the end-users of pulse flours supported previous findings and highlighted the importance of flavor and texture.

### 3.6. Variability

The 21 CPC users were asked questions about variability in pulse flour quality by batch or supplier, universal specifications of pulse flour or the lack thereof, and gluten and lectin concerns (Table 11). Three respondents agreed that pulse flour quality (sometimes) varies from batch to batch, seven disagreed, and ten did not know. Half of the current users indicated pulse flour quality does not vary from batch to batch. One respondent who selected “Sometimes” commented that the quality is “harvest dependent”. In contrast, for variation by suppliers, 11 users agreed that pulse flour quality (sometimes) varies from supplier to supplier. This seems to reflect the current situation in the pulse milling industry, where universal specifications are absent for pulse flours, unlike wheat flour, which results in pulse flour products with varying characteristics milled by the supplier’s own method [25]. Interestingly, however, not everyone agreed that the variation in pulse flour quality affects their product consistency or that having universal specifications is a critical factor for them. Two respondents who indicated that the variation in pulse flours “sometimes” makes it difficult to produce food products with consistent quality commented that it depends on pulse varieties, growing locations, and weather conditions. These comments showed that some users are aware of the effect of pulse varieties and seasonal conditions on flour quality and, thus, on the food product quality made from it. Regarding universal specifications, one participant who selected “Sometimes” commented that it “Depends on the level of incorporation of the pulse flour in the formulas”, suggesting that specifications may not matter as much if only a small percentage of pulse flour is incorporated in other flours. Fifteen respondents indicated that gluten contamination is (sometimes) a concern in pulse flours, but only six indicated that lectins in pulse flours are (sometimes) a concern, similarly to the bean flour impression results (Table 5).

### 3.7. Supply and Logistics

Extrinsic factors such as cost, production, and influence of the COVID-19 pandemic on pulse flour supply were assessed by the 21 CPC users (Table 12). Twelve agreed that there is a supplier that provides them with their pulse flour at a reasonable cost. The percentage of those who agreed were more or less similar for each user type: 63% of the current users, 50% of the previous users, and 56% of the considered users. It was intriguing that seven agreed that freight cost is a critical factor, but five did not. Eight to ten participants either did not know or did not answer to these questions about supply and logistics. More than half (11) of participants agreed that there is enough supply of pulse flour for their production scale, while none indicated that there is not enough supply. These results suggest that pulse flour supply or cost may not be the main barrier to pulse flour use for food manufacturers. The COVID-19 pandemic saw a surge in sales of pulse products [50], but only three indicated pulse flour was less available, while seven indicated that the availability is higher or the same.

### 3.8. Collaboration with Pulse Breeders

Participants were asked if they would be interested in providing opinions to university researchers and plant breeders about how pulse flours can be improved. In total, 9 of the 74 respondents answered yes. Two other respondents left comments that they do not know enough about pulse flours to provide useful feedback or that someone else in the company might be willing to assist. Most of the “yes” respondents were from a single food manufacturer location. Such collaboration will be useful in developing pulse varieties that meet the end-users’ needs and will increase pulse flour adoption. For example, an industry partner was involved in the lab-scale evaluation of pulse flours for crackers, and a selected pulse type (chickpea) was further tested on a commercial scale at the company [51].

## 4. Limitations of the Study

The response rate for the email survey solicitation was low at 1.06% (109/10,311). Throughout the study development, in interviews with food manufacturers and pilot data collection, employees often expressed concerns about sharing corporate information. The low response rate may be due to the industry’s hesitation in answering questions about their production and research activities that are proprietary. Industry professionals are a difficult population to obtain information from, even though it was clearly explained in the invitation email and the survey front page that this survey is for academic research and that no identifiable information will be published. One invitee selected “Not comfortable answering questions about the company activities” as the reason not to participate. Another invitee to the preliminary interview stated that they cannot participate because the questions are [asking for information that is] mostly confidential for them. A similar observation was made in another pulse products innovation study. Only two firms filed a patent on their new method of pulse processing (which will be published), and all others chose to hold it as a trade secret [29].

Despite pilot testing and expert content review, the survey form may have contained questions or choices that participants did not understand or interpret as intended. Words such as flavor, texture, and dough handling properties could mean various attributes depending on products.

## 5. Conclusions

An online survey was administered to 75 food industry professionals whose firms produce food products from regular wheat and/or gluten-free flours. The majority of respondents did not use pulse flours. Yeast breads were the most selected product type for which they are using, used, considered using, or would be interested in using pulse flours. Chickpea and pea were the top two pulse flours selected. For the industry personnel who looked for technical information on pulses, scientific associations, both industry and academic, and open-access journals were the main sources. Regarding bean flour in particular, increasing publicity and improving flavor and functionality may be key improvements for inclusion in flour-based food products. Lectin and gluten contaminations were less selected by respondents as concerns. Market demand are likely to be just as important as or more important for continued use of pulse flours than intrinsic characteristics. According to the 21 CPC users, texture, appearance, and uniformity were the three most selected satisfactory characteristics of products using their pulse flour. Ten stated that there were challenges, but five stated there were no challenges. Flavor, texture, dough handling properties, mouthfeel, and volume were the most selected challenges. More than half of the CPC users agreed that pulse flour quality (sometimes) varies from supplier to supplier; however, opinions were mixed about universal specifications for pulse flours and the effect of the variation on the consistency of product quality. Extrinsic factors such as pulse flour supply or cost may not be a main problem to pulse flour users. In summary, flavor, texture, lack of gluten, and market demand were important factors in pulse flour use among food industry professionals who participated in this survey. Individual characteristics that need improvements for specific pulse flour and product type should be studied in future research.

## Figures and Tables

**Table 1:** Standard Industrial Classification (SIC) codes of food manufacturing firms targeted in this study.

SIC Code	Product Category
2045	Prepared Flour Mixes and Doughs
2051	Bread and Other Bakery Products
2052	Cookies and Crackers
2053	Frozen Bakery Products
2096	Potato Chips/Corn Chips/Similar Snacks
2098	Macaroni, Spaghetti, Vermicelli, and Noodles

**Table 2:** Participants’ interest in pulse flour type by user category.

	Total Count	Current (n = 8) ^a^	Previous (n = 4)	Considered (n = 9)	Never (n = 54)
		%			
Chickpea	13	38	0	56	9
Pea	13	63	50	33	6
Bean	2	0	25	11	0
Fava	1	0	25	0	0
Lentil	1	0	0	0	2
Do not know which is good	37	0	0	0	69
Not interested	7	0	0	0	13
No answer	1	0	0	0	2
Total	75	100	100	100	100

^a^ n indicates the number of participants in each type of users.

**Table 3:** Participants’ interest in food product type by user category.

	Total Count	Current (n = 8) ^a^	Previous (n = 4)	Considered (n = 9)	Never (n = 54)
		%			
Yeast breads, buns, and rolls	21	25	50	22	28
Cookies, bars, and crackers	11	0	0	11	19
Not interested	11	0	0	0	20
Quick breads such as muffins	9	25	25	0	11
Snack foods such as chips, puffs	6	13	25	33	2
Other	4	0	0	0	7
Pastries and pies	4	0	0	0	7
Plant-based meat alternatives	4	25	0	22	0
Cakes	3	0	0	11	4
Thickening agents for soups, gravies	2	13	0	0	2
Total	75	100	100	100	100

^a^ n indicates the number of participants in each type of users.

**Table 4:** Pulse flour type and product type that participants are using, previously used, considered using, or interested in using. A total 14 of “Not interested” or no answers were excluded from this table.

	Do Not Know Which Is Good	Chickpea	Pea	Bean	Fava	Lentil	Total
Yeast breads, buns, and rolls	9	4	4	0	1	1	19
Cookies, bars, and crackers	9	0	2	0	0	0	11
Quick breads such as muffins	4	3	2	0	0	0	9
Snack foods such as chips, puffs	1	2	1	2	0	0	6
Other	2	1	1	0	0	0	4
Plant-based meat alternatives	0	2	2	0	0	0	4
Cakes	2	1	0	0	0	0	3
Pastries and pies	3	0	0	0	0	0	3
Thickening agents for soups, gravies	1	0	1	0	0	0	2
Total	31	13	13	2	1	1	61

**Table 5:** Participants’ impression on bean flour by user category.

	Total	Current (n = 8) ^a^	Previous (n = 4)	Considered (n = 9)	Never (n = 54)
	Count	%			
Do not know about bean flour	37	25	25	33	57
Flavor is a challenge	22	63	0	67	20
Functionality is a challenge	21	38	25	44	24
Market demand for bean flour is low	13	38	25	11	15
More expensive than other GF flours	10	25	25	0	13
Do not know how to use bean flour	9	13	0	22	11
Gluten contamination is a concern	5	13	0	11	6
Lack of specification for bean flour is a challenge	3	0	0	0	6
Not available from a local source	3	0	25	0	4
Other	3	0	0	0	6
Lectins in bean flour are a concern	1	0	25	0	0

^a^ n indicates the number of participants in each type of users.

**Table 6:** The combinations of product and pulse flour that the 21 current, previous, or considered (CPC) users selected. Counts more than one are shown in parentheses.

Count	Product	Pulse Flour Type
6	Yeast breads, buns, and rolls	Pea (3), Chickpea (2), Fava
5	Snack foods such as chips, puffs	Bean (2), Chickpea (2), Pea
4	Plant-based meat alternatives	Pea (2), Chickpea, Other
3	Quick breads such as muffins	Pea (2), Chickpea
1	Cakes	Chickpea
1	Cookies, bars, and crackers	Pea
1	Thickening agents for soups, gravies	Pea

**Table 7:** Reasons for currently using pulse flours selected by eight current users.

Reason for Using	Count	(%)
Driven by marketing and trends	5	63
Protein content	3	38
Functional characteristics of the pulse flour	3	38
Environmental sustainability	2	25
Gluten-free attributes	2	25
Health benefits to consumers	1	13
Improved protein quality	1	13
Other	1	13
Cost-saving	0	0

**Table 8:** Combination of product type and pulse flour type by the presence or absence of challenges in production. Counts more than one are shown in parentheses.

Challenges	Product Type	Pulse Flour Type	Count	Total Count
Yes, there is (are) challenge(s)				10
	Yeast breads, buns, and rolls	Chickpea (2), Pea (2), Fava	5	
	Snack foods such as chips, puffs	Bean, Chickpea	2	
	Plant-based meat alternatives	Chickpea, Pea	2	
	Cookies, bars, and crackers	Pea	1	
No, there was no challenge				5
	Quick breads such as muffins	Chickpea, Pea	2	
	Snack foods such as chips, puffs	Bean, Chickpea	2	
	Thickening agents for soups, gravies	Pea	1	

**Table 9:** Satisfactory product qualities produced using the pulse flours that the CPC users selected.

	Total Count	Current (n = 8) ^a^	Previous (n = 4)	Considered (n = 9)
		%		
Texture	11	63	75	33
Appearance	9	63	50	22
Uniformity	8	25	75	33
Color	7	63	25	11
Flavor	7	38	50	22
Mouthfeel	7	38	50	22
Cooking qualities	6	25	100	0
Dough handling properties	6	25	50	22
Extrusion properties	4	25	25	11
Mixing properties	4	25	25	11
Surface smoothness	4	25	25	11
Product yield	3	13	50	0
Shape	3	0	50	11
Shelf life	3	0	50	11
Mitigating cross-contamination	1	0	25	0
Volume	1	0	25	0
Other	1	13	0	0

^a^ n indicates the number of participants in each type of users.

**Table 10:** Challenging product qualities produced using the pulse flours that the 10 CPC users selected who reported they encountered challenges.

	Total Count	Current (n = 3) ^a^	Previous (n = 2)	Considered (n = 5)
		%		
Flavor	8	100	100	60
Texture	6	0	50	100
Dough handling properties	4	33	50	40
Mouthfeel	4	0	100	40
Volume	4	0	100	40
Appearance	3	0	100	20
Cooking qualities	3	33	50	20
Mixing properties	3	33	0	40
Shape	2	33	50	0
Shelf life	2	33	50	0
Color	1	0	50	0
Extrusion properties	1	0	0	20
Mitigating cross-contamination	1	33	0	0
Size	1	0	50	0
Surface smoothness	1	0	0	20
Uniformity	1	33	0	0
Other	1	33	0	0

^a^ n indicates the number of participants in each type of users.

**Table 11:** The CPC users’ opinions on pulse flour quality variability, universal specification, gluten contamination, and lectins.

	Total	Current (n = 8) ^a^	Previous (n = 4)	Considered (n = 9)
	Count	%		
Pulse flour vary from batch to batch?				
Yes	2	0	25	11
Sometimes	1	13	0	0
No	7	50	25	22
Do not know	10	38	25	67
No answer	1	0	25	0
Pulse flour vary from supplier to supplier?				
Yes	10	75	25	33
Sometimes	1	0	0	11
Do not know	4	0	50	22
We only purchase from one supplier ^b^	6	25	25	33
Does variation in pulse flour quality affect product quality consistency?				
Yes	6	13	25	44
Sometimes	2	25	0	0
No	5	25	50	11
Do not know	6	25	0	44
No answer	2	13	25	0
Is having universal specifications a critical factor in utilizing the pulse flour?				
Yes	6	25	50	22
Sometimes	4	13	50	11
No	4	25	0	22
Do not know	7	38	0	44
Is gluten contamination a concern?				
Yes	14	88	25	67
Sometimes	1	0	0	11
No	5	0	75	22
Do not know	1	13	0	0
Are lectins in pulses a concern?				
Yes	4	13	25	22
Sometimes	2	0	25	11
No	10	63	0	56
Do not know	5	25	50	11

^a^ n indicates the number of participants in each type of users. ^b^ The question about variation by suppliers had an additional choice: “We only purchase from one supplier”.

**Table 12:** The CPC users’ opinions on the supply and logistics of pulse flours and the availability of pulse flours during the pandemic of COVID-19.

	Total Count	Current (n = 8) ^a^	Previous (n = 4)	Considered (n = 9)
		%		
Are there suppliers who provide your pulse flour at a reasonable cost?				
Yes	12	63	50	56
No	1	0	25	0
Do not know	8	38	25	44
Is freight cost a critical factor?				
Yes	7	38	25	33
No	5	25	0	33
Do not know	6	38	50	11
No answer	3	0	25	22
Is there enough supply of the pulse flour for your production scale?				
Yes	11	88	25	33
Do not know	10	13	75	67
Has the availability of the pulse flour changed since March 2020 due to the pandemic of COVID-19?				
More available	2	25	0	0
Same as before	5	25	0	33
Less available	3	13	25	11
Do not know	11	38	75	56

^a^ n indicates the number of participants in each type of users.

## Data Availability

The data presented in this study are not available due to potential issues of confidentiality with the respondents.

## Supplementary Materials

The following supporting information can be downloaded at: https://www.mdpi.com/article/10.3390/foods11142146/s1, Table S1: Job titles targeted in the second distribution of the survey.
