# Supplementary material for: Food Industry Views on Pulse Flour—Perceived Intrinsic and Extrinsic Challenges for Product Utilization
Source: Foods. 2022 Jul 20;11(14):2146. doi: 10.3390/foods11142146 (PMC9319253; doi:10.3390/foods11142146)
Supplement: Supplementary file 1 [file foods-11-02146-s001.zip › foods-1791565-supplementary.pdf]

Supplementary Table:

Table S1. Job titles targeted in the second distribution of the survey.

Business Development
CEO
Chief Marketing Officer
Director
Engineering/Technical
Executive
Executive Director
Executive Officer
Executive Vice President
General Manager
International
Manager
Manufacturing
Manufacturing Executive
Marketing
Marketing Executive
Operations
Operations Executive
Owner
President
Principal
Purchasing Executive
Regional Manager
Sales
Sales Executive
Senior Vice President
Site Manager
Vice President
